# Role of Tpm Isoforms Produced by the *TPM4* Gene in the Regulation of Actin Filament Dynamics by Cofilin

**DOI:** 10.3390/biom15081206

**Published:** 2025-08-21

**Authors:** Svetlana G. Roman, Victoria V. Nefedova, Alexander M. Matyushenko

**Affiliations:** Research Center of Biotechnology of the Russian Academy of Sciences, 119071 Moscow, Russia; svetabaj@gmail.com (S.G.R.); victoria.v.nefedova@mail.ru (V.V.N.); ammatyushenko@mail.ru (A.M.M.)

**Keywords:** actin-associated proteins, tropomyosin, actin, cofilin, actin dynamics, actin severing, actin polymerization/depolymerization

## Abstract

The actin cytoskeleton determines a huge number of intracellular processes, as well as maintaining the cell shape, transport, formation of intercellular contacts, etc. The actin cytoskeleton’s function is largely determined by actin-binding proteins. Here, the mutual influence of two actin-binding proteins, cofilin (cof) and tropomyosin (Tpm), is studied. In the present work, using various biochemical approaches, we reveal the effects of two *TPM4* gene-derived isoforms (Tpm4.1 and Tpm4.2) in the presence of cofilin-1 and cofilin-2. The cofilin severing activity was estimated in F-actin and Tpm/F-actin complexes using viscosity measurements and electron microscopy. Both cofilins prompted the disassembly of F-actin filaments with Tpms attached to them, and the Tpm4.2 isoform demonstrated a better protective effect. We also estimated the ability of cofilin-1 and cofilin-2 to displace Tpms from actin filaments by using the co-sedimentation method. Both cofilin isoforms efficiently displaced Tpm4.1 and Tpm4.2 and bound to actin filaments. Both Tpms decreased the initial rate of actin polymerization in the presence of cofilin-1 and cofilin-2. Overall, we can assume that Tpm4.1 and Tpm4.2 do not affect the binding of cofilin to actin filaments, which may be important for cofilin to exhibit its severing activity and lead to the remodeling of the actin cytoskeleton.

## 1. Introduction

Actin is the most common protein in cells. The unique shape of a cell and its ability to move are largely determined by the actin cytoskeleton [1,2,3]. Actin is a key player in many cellular processes, such as cytokinesis, vesicle formation, transport of cellular organelles, maintenance of the mechanical stability of the cell, and participation in the formation of cell contacts [4,5,6,7,8]. A large number of partner proteins play a significant role in the actin cytoskeleton’s functions [9,10]. An interesting and important task to understand the functioning of the cytoskeleton is to study how a specific function of the actin filaments is determined at a certain place and at a certain time in the cell, as well as how this process is regulated.

Actin exists in two forms in the cell: monomeric G-actin and polymeric F-actin. The maintenance of these two states is ensured by the processes of polymerization and depolymerization. Maintaining the actin pool in two different states is extremely important for the cell, since it provides the necessary dynamics of the formation of cellular structures and cellular activity [11]. Filamentous actin, in turn, can form both stable structures and dynamically changing ones [12,13,14]. Changes in its architecture and dynamic state are determined by actin-binding proteins. The key participants in this process are proteins belonging to the ADF/cofilin and tropomyosin families [15,16,17].

ADF (destrin) and two isoforms of cofilin belongs to the ADF/cofilin family of proteins and are considered to be destabilizing factors for filamentous actin [18]. They can attach to the old ADP–actin filament segments, change the conformation of the actin filament at the point of attachment, and introduce breaks in the actin thread [19,20,21]. Cofilin is able to change the twisting of actin filaments [22] and has also been shown to be involved in the destabilization of interaction contacts at the adjacent actin interface [23,24,25,26]. In addition, cofilin has been found to have depolymerizing activity and can also participate in the sequestration of actin monomers [27,28]. All this undoubtedly makes it a key participant in the regulation of actin dynamics in the cell.

However, cofilin has also been shown to have activity that is not associated with actin filament destabilization, but rather promotes the formation of stable structures. Thus, an excess of cofilin relative to actin, on the contrary, stabilized filamentous actin [29,30]. It has also been found that the cross-linking of two cofilin monomers via a disulfide bond can lead to the appearance of cross-links in two adjacent actin filaments, which leads to the stabilization of actin rods in cells [31,32]. These findings indicate that the role of cofilin in cells can be much broader and depends on many factors.

Two isoforms of cofilin have been found in animal tissues: cofilin-1 and cofilin-2 [33]. These are proteins of a similar molecular weight and structure. The main isoform of cofilin expressed in muscle tissues is cofilin-2 [34,35,36], while in non-muscle tissues, cofilin-1 is mainly expressed [37]. The expression of these proteins can be significant and reach micromolar concentrations [38]. It is worth noting that the expression of cofilin-1 has been detected in muscle tissues as well, and cofilin-2 is also expressed in smaller amounts in non-muscle tissues [39]. This indicates that different cofilin isoforms may have specific functions in all cells.

Tropomyosin is the opposite of cofilin in terms of its effect on actin filaments. Tropomyosin is a large family of proteins expressed in all animal tissues [40]. Tropomyosin is believed to be involved in the differentiation of actin filaments’ functions [41]. It is attached to actin filaments along the entire actin surface and forms an interface for interaction with actin-binding proteins. The high diversity in the formation of functionally different actin microfilaments is achieved by the production of many tropomyosin isoforms [42]. Such a role for tropomyosin is confirmed by its localization in cells, in which patterns of preferential localization for various tropomyosin isoforms can be found [43,44]. Interestingly, similar differentiation is achieved in plant cells by the production of a large number of actin isoforms [45].

Four genes producing about 40 different isoforms of tropomyosin have been found in mammalian cells [40,42]. They are usually divided by their molecular weight into “short” and “long” isoforms [40,46]. The “short” isoforms cover six actin monomers with one tropomyosin molecule, while the high-molecular-weight forms cover seven monomers [47]. Since the majority of actin filaments in differentiated cells are covered by tropomyosin [48] and it has been shown to play a key role in the stabilization of actin filaments and the formation of various actin structures [46,47,49], it also plays a key role in the regulation of the actin filaments’ dynamics [50]. The study of the interaction of tropomyosin with proteins in the cofilin family is an interesting task in this regard.

It is generally accepted that these two proteins act as antagonists of each other [49,51,52]. However, a number of studies have shown that tropomyosin increases the destabilizing effect of cofilin [43,53]. Thus, this allows us to conclude that the combined action of these proteins depends on their composition [54,55]. For example, the non-muscle isoforms Tpm1.6 and Tpm1.8 effectively prevented actin filament depolymerization [56,57]. Muscle isoforms of tropomyosin (Tpm1.1, Tpm3.12) also prevented actin filament disassembly [58,59]. At the same time, short isoforms (Tpm3.1, Tpm3.2, Tpm3.4, and Tpm4.2) were not as good at protecting actin filaments from the effects of cofilin [55,56].

Most studies of tropomyosin’s interaction with cofilin have been performed on the tropomyosin isoforms produced by the *TPM1* and *TPM3* genes. Much less research has been performed on the isoforms produced by *TPM4*. This gene encodes only two forms of tropomyosin: Tpm4.1 and Tpm4.2 [42]. The least studied of these isoforms is Tpm4.1. The expression of this isoform has been shown in fish muscle cells and mammalian epithelial tissue [60,61]. The disruption of this isoform’s expression in mammalian epithelial tissue resulted in cancer progression [61]. A slightly more studied isoform is Tpm4.2, which is expressed in many tissues [47,62,63]. Tpm4.2 is known as the main isoform in the postsynaptic space in neurons [63], and along with isoform Tpm4.1 it has also been found in the hearts of fish [60]. Tpm4.2 has been shown not to interfere with the destabilizing effect of cofilin-1 on F-actin [55].

These two isoforms can potentially coexist with both cofilin isoforms in tissues, making them an interesting case to study. Considering the fact that Tpms (including the products of the TPM4 gene) determine the formation of specific microfilaments in the cell, their interaction with cofilin, a protein responsible for dynamic changes in the actin cytoskeleton, may be of decisive importance for the formation of specific cytoskeletal compartments. In our work, using various biochemical approaches, we studied the interaction of the Tpm4.1 and Tpm4.2 tropomyosin isoforms with cofilin-1 and cofilin-2.

## 2. Materials and Methods

### 2.1. Protein Expression and Purification

pET-23a+ plasmids with coding sequences for human Tpm4.1 and Tpm4.2 were obtained using the gene synthesis service provided by Evrogen (Moscow, Russia). Tpm4.1 and Tpm4.2 were expressed in the *E. coli* strain C41 through the induction of 1 mM IPTG during synthesis for 4 h at 37 °C. After expression the cells were collected by centrifugation and subjected to rough extraction. The cells were destroyed by sonication for 10 min of pure time on ice. The cell lysate was heated to 85 °C for 6 min in the presence of 2 M NaCl. Denatured proteins were removed by centrifugation. Tropomyosin was precipitated from the supernatant by adding sodium acetate at pH 4.8 to a final concentration of 100 mM. The precipitate was dialyzed in a 50 mM Tris-HCl buffer at pH 8.0 overnight. The Tpm proteins were purified using standard methods as described previously [64]. All the Tpm species had an Ala-Ser extension at the N-termini to imitate the naturally observed acetylation [65].

The human cofilin-1 and cofilin-2 were recombinant proteins. The coding sequences for cofilins were obtained from the SH-SY5Y cell line by reverse transcription with Mint reverse transcriptase (Evrogen, Moscow, Russia). The reverse primer for cofilin-1, 5’-TCACAAAGGCTTGCCCTCCAGG-3’, and the reverse primer for cofilin-2, 5’-TTATAATGGTTTTCCTTCAAGTGAAACTACTACAT-3’, were used to obtain the first DNA strand. The cds of cofilin-1 and cofilin-2 were first cloned to the pAL2-T vector (Evrogen, Moscow, Russia) and then cloned between NdeI and EcoRI restriction sites of the pET-23a( + ) vector. The 6x-His-tag was added using a Q5 site-directed mutagenesis kit (NEB, Ipswich, MA, USA) with the following forward primers: 5’-ATG CAT CAC CAT CAC CAT CAC CTG GAA GTG CTG TTT CAG GGC CCG ATG GCC TCC GGT GTG GCT GTC TCT-3’ for cofilin-1 and 5’-ATG CAT CAC CAT CAC CAT CAC CTG GAA GTG CTG TTT CAG GGC CCG ATG GCT TCT GGA GTT ACA GTG AAT GAT GAA G-3’ for cofilin-2. The reverse primer for both cofilins was 5’-ATG TAT ATC TCC TTC TTA AAG TTA AAC AAA ATT ATT TCT AG-3’. All plasmids were verified by sequencing.

Recombinant cofilin-1 and cofilin-2 were expressed in the *E. coli* strain C41. Protein expression was induced with 1 mM IPTG and continued at 30 °C overnight. After that the bacteria were lysed in a 50 mM HEPES-NaOH buffer at pH 7.3, containing 300 mM NaCl and 20 mM imidazole. The cofilins were purified using metal affinity chromatography on a HisTrap HP 5 mL (GE Healthcare, Uppsala, Sweden) column. The fractions with the highest amounts of cofilin were collected, concentrated to 5 mL using a JetSpin Centrifugal Filter (BIOFIL, Madrid, Spain), and further purified by gel filtration on a HiLoad 16/600 Superdex 200 pg column (GE Healthcare, Uppsala, Sweden). The purified proteins were dialyzed against a 50 mM HEPES-NaOH buffer at pH 7.3, containing 150 mM NaCl, 0.1 mM PMSF, and 14 mM β-ME. The purity of the cofilin preparations was at least 98%. The protein concentration was determined spectrophotometrically using the following ε^1%^ values at 280 nm: 7.8 cm^−1^ for cofilin-1 and 9.85 cm^−1^ for cofilin-2.

G-actin was extracted from rabbit skeletal muscle acetone powder as described in [66]. F-actin was polymerized by adding 2 mM MgCl_2_ and 100 mM KCl.

### 2.2. Protein Labeling and Preparation for Fluorescence Applications

The actin was labeled with N-(1-pyrene)-iodoacetamide (CHEM-IMPEX, Wood Dale, IL, USA) as described in [67] with minor modifications. A total of 2 mL of the freshly obtained G-actin (5 mg/mL) and 10 mM DTT were dialyzed overnight against three changes of a 25 mM Tris-HCl buffer at pH 7.5, containing 100 mM NaCl, 2 mM MgCl_2_, 0.3 mM ATP, and 3 mM NaN_3_. After this, the polymerized actin was transferred to Amber Eppendorf tubes. N-(1-Pyrene)-iodoacetamide dissolved in dimethyl sulfoxide was added to the F-actin to a final concentration of 0.22 mM and incubated for 26 h at 4 °C with constant stirring. The labeled F-actin was then dialyzed for 48 h against three changes of buffer G, a 2 mM Tris-HCl buffer containing 0.1 mM ATP, 0.1 mM CaCl_2_, and 1 mM NaN_3_, at pH 8.0. Depolymerized actin was centrifuged at 100,000× *g* for 2 h at 4 °C, and the upper fraction of the supernatant was collected. The excess labels were removed using NAP-25 columns (GE Healthcare, Altrincham, UK).

### 2.3. Viscosity Measurements

The viscosity was measured in a 0.5 mL capillary using an AMVn falling ball microviscometer (Anton Paar, Ashland, VA, USA) as described previously in [68]. To determine the exact viscosity of the samples, a DMA 4500 densitometer (Anton Paar, VA, USA) was used to estimate the density of the protein solutions before the viscosity measurements. All the experiments were performed with 10 µM F-actin and 3.3 µM Tpm in a 30 mM HEPES-NaOH buffer at pH 7.5, containing 100 mM NaCl and 1 mM DTT. Cofilins were added to the samples to a final concentration of 1 µM, and the viscosity of the solutions was measured 60 min after addition. The temperature of all the solutions was 20 °C. The measurements were performed at least three times for each sample.

### 2.4. Actin Polymerization Assay

The actin polymerization was observed using a Cary Eclipse fluorescence spectrophotometer (Varian Australia Pty Ltd., Mulgrave, VIC, Australia). The experiments were performed using pyrene-labeled G-actin. To measure the actin polymerization, the fluorescence changes were monitored at λ_ex_ = 340 nm and λ_em_ = 407 nm at 25 °C. Polymerization of 2.5 μM G-actin in a 10 mM HEPES-NaOH buffer at pH 7.5, containing 100 mM NaCl, 2.5 mM Mg^2+^, and 0.2 mM ATP, was initiated by adding an aliquot of an equimolar G-actin/pyrenyl–G-actin mixture to a pre-incubated buffer containing or lacking 0.25 μM cofilin, 1 μM Tpm, or both. The measurements were performed 3–6 times for each case and average curves were obtained.

### 2.5. Binding of Cofilin to F-Actin and Displacement of Tropomyosin from Actin Surface

To estimate the cofilin binding activity and displacement of tropomyosin from the actin surface, we used a co-sedimentation assay. Samples containing 9.6 µM F-actin and 5 or 10 µM Tpm were incubated for 30 min in a 30 mM HEPES-NaOH buffer (pH 7.5) with 100 mM NaCl, 2 mM MgCl_2_, and 1 mM DTT. Then, cofilin was added to the samples in concentrations ranging from 2.5 to 30 µM, and they were incubated for 40 min. The obtained probes were precipitated at 100,000 *g* for 1 h. The pellet and supernatant fractions were analyzed using SDS-PAGE. The protein bands were analyzed using Image J2 software (Scion, Frederick, MD, USA). The measurements were repeated three times for all the samples.

### 2.6. Rhodamin–Phalloidin Fluorescence Assay

To detect the structural changes in the F-actin strands, we performed a rhodamine–phalloidin fluorescence decay assay. The experiments were carried out as described in [56,69] with minor alterations. Rhodamine–phalloidin and phalloidin were mixed at a molar ratio of 1:99 and added to F-actin at a total equimolar concentration. F-actin with phalloidin was incubated overnight. The labeled F-actin was then dialyzed against a 30 mM HEPES-NaOH buffer at pH 7.5, containing 100 mM NaCl and 1 mM DTT. The labeled F-actin was pre-incubated with or without Tpms for 1 h before the measurements, and the cofilins were added in a 2:1 molar ratio to F-actin. To measure the release of rhodamine–phalloidin from F-actin, the fluorescence changes were monitored at λ_ex_ = 520 nm and λ_em_ = 575 nm using a Cary Eclipse fluorescence spectrophotometer (Varian Australia Pty Ltd., Mulgrave, VIC, Australia) at 25 °C.

### 2.7. Determination of F-Actin Lengths by Electron Microscopy

The samples of F-actin and F-actin incubated with Tpms and cofilins were studied by transmission electron microscopy. The samples were prepared as described in Section 2.3. A total of 10 μL of the samples were spotted on carbon support films for 30 s and negatively stained with 10 μL of 1% uranyl acetate for 30 s. The resulting grids were observed using a JEM 1400 electron microscope (JEOL, Tokyo, Japan) operated at 80 kV. To quantify the F-actin filaments’ lengths, 101−152 filaments from each sample were measured by using ImageJ 1.53c software (Scion, Frederick, MD, USA). The lengths of short filaments were measured directly in the frame that contained them. The lengths of longer actin filaments were measured either at a lower magnification or using “overlaid” frames obtained at a higher magnification. A two-sample Kolmogorov–Smirnov test was applied to determine whether there were significant differences.

## 3. Results

### 3.1. Cofilin Severing and Depolymerizing Activity Determined Through Viscosity Measurements

**Figure 1:**
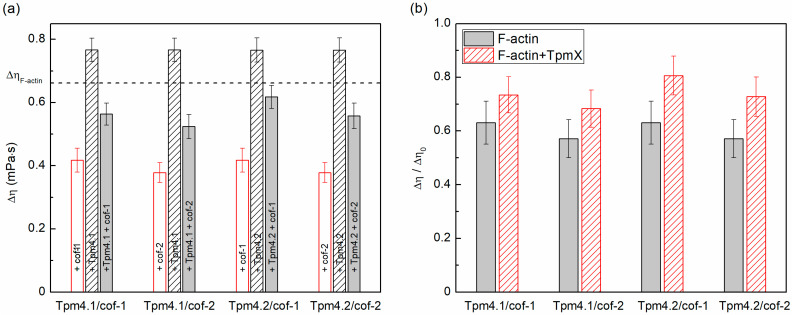
The effect of cofilin-1 and cofilin-2 on the viscosity of the solutions of actin filaments covered by Tpm4.1 or Tpm4.2. All values are presented as the mean ± the 95% confidence interval. (**a**) Absolute values of the excess viscosity over the buffer viscosity (Δη) for different actin filament solutions. The dashed line indicates the mean excess viscosity value obtained for F-actin. (**b**) The ratio of the excess viscosity of the F-actin solutions in the presence of different cofilin isoforms (Δη) and the viscosity of the corresponding solutions without cofilin (Δη_0_). To determine the significance of the differences in the viscosities, Student’s *t*-test was used (n = 16). The viscosity of the samples in F-actin +/-cofilin, F-actin +/-Tpm, F-actin + cofilin +/-Tpm, and F-actin + Tpm +/-cofilin differed significantly (*p* < 0.5).

We evaluated the severing activity of cofilins in F-actin and Tpm/F-actin complexes using viscosity measurements. The excess viscosity values of the buffer solutions containing only Tpms were 0.016 ± 0.001 mPa∙s and 0.012 ± 0.001 mPa∙s for 3.3 mM Tpm4.1 and Tpm4.2, respectively. The average excess viscosity value was 0.66 ± 0.04 mPa∙s for F-actin and 0.77 ± 0.04 mPa∙s for the F-actin−Tpm4.1 and F-actin−Tpm4.2 mixtures (see Table 1). As can be seen, the overall viscosity of the mixtures could not be obtained by simply adding together the excess viscosity values of the individual components and significantly exceeded them. This indicates that the viscosity of F-actin solutions depends on the length of the F-actin threads: in the presence of these Tpms, they are likely to be longer. Actin filaments are shortened under the action of cofilins, which was reflected in a decrease in the solution viscosity. The experimental results are presented in Figure 1 and Table 1. The absolute solution viscosity values in the four sample combinations significantly differed from each other (tested with Student’s *t*-test, *p* < 0.05, n = 16).

It is clearly visible that both cofilins led to the disassembly of the actin filaments of both Tpm-covered and bare F-actin. It is important to note that the initial viscosity values of the solutions of bare actin and actin covered by Tpm were different from each other. They were higher in the case of the Tpm-covered filaments. To establish the effect of Tpm on the cofilin severing activity, we normalized the values obtained after 1 h incubation with cofilins by the initial viscosity values in each case (Figure 1b). The greatest relative protective effect of Tpm at the indicated concentration ratios was observed in the case of the Tpm4.2 isoform in relation to cofilin-1.

### 3.2. Binding of Cofilin to F-Actin and Displacement of Tpm from Actin Surface

**Figure 2:**
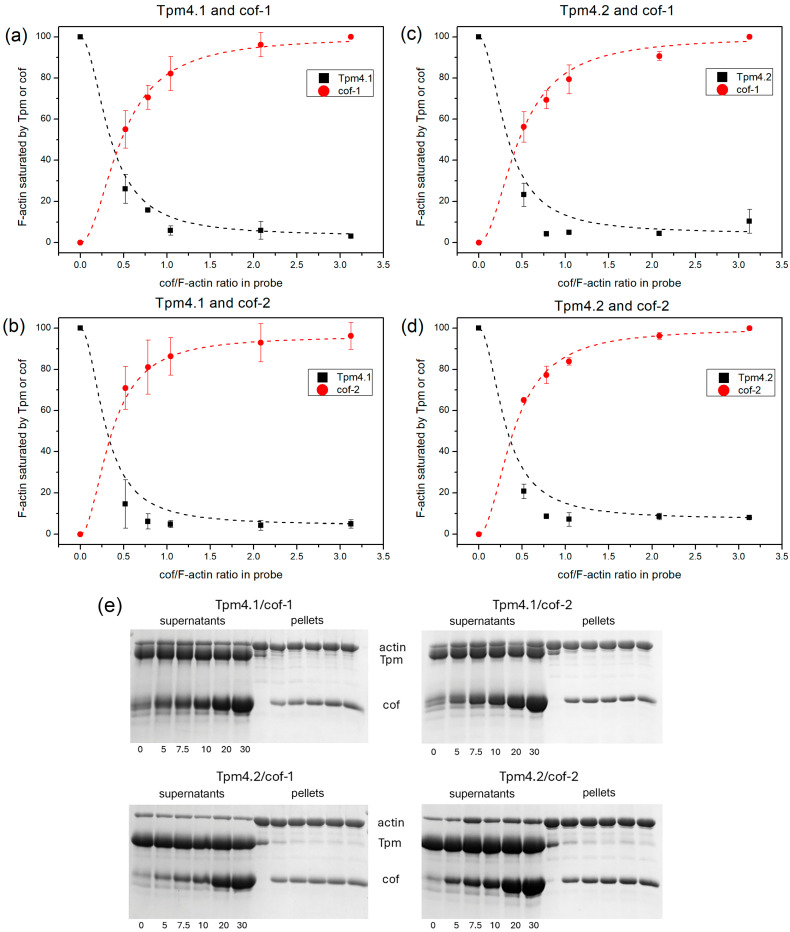
Results of co-sedimentation assay obtained for F-actin reconstructed using various isoforms of Tpm and cofilin: (**a**) F-actin covered by Tpm4.1 and cofilin-1; (**b**) F-actin covered by Tpm4.1 and cofilin-2; (**c**) F-actin covered by Tpm4.2 and cofilin-1; and (**d**) F-actin covered by Tpm4.2 and cofilin-2. Figures show average values obtained from three experiments. All values are mean ± SD. (**e**) Representative gels obtained by SDS-PAGE. [Tpm] = 10 μM; concentration of [cofilin] is indicated under gel lanes. Original images can be found in Supplementary Materials.

Cofilin and Tpm are actin-binding proteins that can compete to bind with the actin surface. We assessed the ability of cofilin-1 and cofilin-2 to bind actin filaments in the presence of Tpm4.1 and Tpm4.2 using the co-sedimentation method. The results are shown in Figure 2.

Both cofilin isoforms efficiently bound to F-actin in the presence of Tpm4.1 and Tpm4.2 (Figure 2, red dots). At the same time, the isoforms of Tpm had virtually no effect on the cofilin binding (there were no significant differences based on the *t*-test). The pattern of saturation of the actin filaments with cofilin did not change depending on which isoform of Tpm F-actin was covered with (Figure 2, red dots).

Also, both cofilin isoforms effectively displaced Tpm from the actin surface (Figure 2, black dots). At 50% saturation of F-actin with cofilin, 50% of the bound tropomyosin was displaced. There was no apparent difference in the displacement of Tpm between the cofilin isoforms.

Saturating concentrations of Tpms were used in this experiment. The *K*_50%_ values measured in the same buffer containing 200 mM NaCl were 1.07 ± 0.07 μM for Tpm4.2 [70] and 3.02 ± 0.22 μM for Tpm4.1 [71], as determined previously. These parameters measured under conditions of weak binding of Tpm to the actin filament were significantly lower than the concentrations used in this experiment.

### 3.3. Rhodamine–Phalloidin Decay Induced by Cofilins

**Figure 3:**
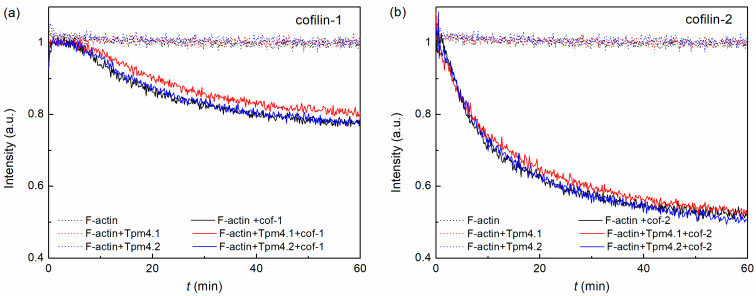
Rhodamine–phalloidin fluorescence decay obtained for F-actin, F-actin/Tpm4.1, and F-actin/Tpm4.2 complexes in absence or presence of cofilin-1 (**a**) and cofilin-2 (**b**)**.** Dotted lines represent curves obtained in absence of cofilin and solid lines those obtained in presence of cofilin molecules.

Structural rearrangements in the F-actin filaments induced by cofilin molecules were estimated using the rhodamine–phalloidin fluorescence decay. We performed these experiments for two isoforms of cofilin in the presence of Tpm4.1 and Tpm4.2 (Figure 3).

Cofilin-1 and cofilin-2 resulted in the displacement of rhodamine–phalloidin in both the presence and absence of Tpm (Figure 3). The displacement rate was higher in the case of cofilin-2 (Figure 3b) compared to cofilin-1 (Figure 3a). It is important to note that both Tpm isoforms did not affect the change in the actin filament conformation in the presence of cofilin-1 or cofilin-2.

### 3.4. Determination of Actin Filaments’ Lengths Through Electron Microscopy Experiments

**Figure 4:**
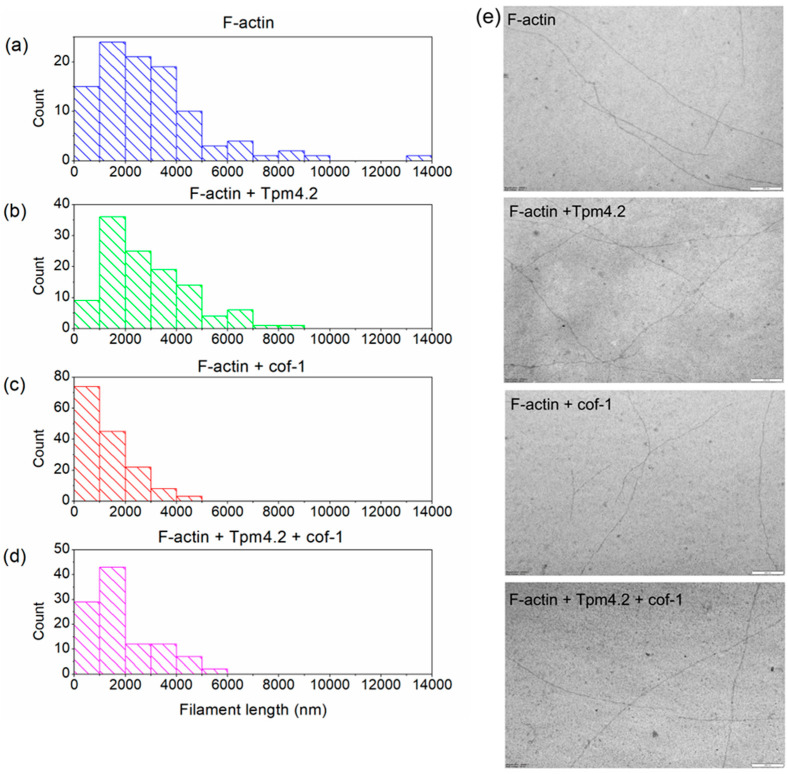
Electron microscopy of bare and Tpm4.2-covered F-actin filaments treated with cofilin-1. Filament length distributions of (**a**) F-actin; (**b**) F-actin with Tpm4.2; (**c**) F-actin with cofilin-1; and (**d**) F-actin with both Tpm4.2 and cofilin-1. Conditions were 0.2 μM F-actin, 0.83 μM Tpm4.2, and 0.02 μM cofilin-1. (**e**) Electron micrographs of negatively stained samples used for quantification. Bar is 200 nm. To determine significant differences Kolmogorov–Smirnov test was applied. All samples, except those of F-actin and F-actin/Tpm4.2 pair, significantly differed from each other, with *p* ≤ 0.05.

Since Tpm4.2 had a protective effect on the severing action of cofilin, we decided to measure the lengths of the actin filaments in the presence of this pair of proteins. To determine the real length of the actin filaments, we used electron microscopy. The results are shown in Figure 4.

The length distributions of bare F-actin and Tpm4.2-covered F-actin were similar to each other. The average length of the filaments was up to 5000 nm (Figure 4a,b,e). However, single filaments with a length from 5000 to 10000 nm were observed in the case of Tpm4.2-covered actin, and even a length of 14000 in the case of bare actin (Figure 4a,b). It should also be noted that actin covered with Tpm4.2 had a larger proportion of filaments with an average length of 1000 to 2000 nm than bare actin (Figure 4a,b).

Cofilin-1, in turn, changed the length of the actin filaments and made them shorter in both bare and Tpm-covered actin (Figure 4c,d). In the case of cofilin acting on bare F-actin, most of the actin filaments had a length in the range from 0 to 3000 nm, with a major fraction with a length of up to 1000 nm (Figure 4c). In the presence of Tpm4.2, most actin filament lengths fell within a range from 0 to 4000 nm, with the main fraction constituting length values varying from 1000 to 2000 nm (Figure 4d).

### 3.5. Effect of Tropomyosin and Cofilin on Actin Polymerization Rate

**Figure 5:**
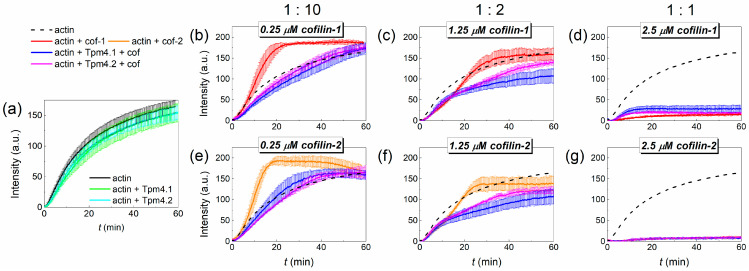
Results of an actin polymerization assay in the presence of different tropomyosin (Tpm4.1 and Tpm4.2) and cofilin (cof-1 and cof-2) isoforms. Effect of Tpm4.1 and Tpm4.2 on the actin polymerization rate in the absence (**a**) and presence of cofilin-1 (**b**–**d**) and cofilin-2 (**e**–**g**) at 1:10 (**b**,**e**), 1:2 (**c**,**f**), and equimolar (**d**,**g**) ratios to actin. The curves are presented as the mean of 3 repeats ± the SD. The upper row shows the ratio of the molar concentrations of the cofilins to that of actin in the corresponding panels below. The dashed curve in panels (**b**–**g**) corresponds to the control polymerization curve of actin alone.

One of the most important properties of actin is its ability to polymerize. To study the effect of cofilin on this process in the presence of Tpm4.1 and Tpm4.2 isoforms, we observed the fluorescence of pyrene-labeled G-actin (Figure 5).

Actin polymerization was accelerated by any of the cofilins at a concentration ten times lower than the actin concentration (Figure 5b,e). However, an increase in the cofilin concentration led to a slowdown in actin polymerization (Figure 5c,f). At an equimolar ratio, the fluorescence signal was very low that is associated with fluorescence quenching by cofilin (Figure 5d,g). Both Tpm4.1 and Tpm4.2 did not change the actin polymerization rate within the error limits (Figure 5a). The combined effect of cofilin and Tpm was more complex. With an excess of actin over cofilin, Tpm4.1 and Tpm4.2 decreased the initial rate of actin polymerization in the presence of both cofilin-1 and cofilin-2. However, if, at a lower concentration of cofilins, after some time the polymerization amplitude reached and even exceeded the characteristic amplitude of actin polymerization without the addition of the actin-binding proteins (Figure 5b,e), then with an increase in the concentration of cofilin the signal amplitude remained lower than that of the control (Figure 5c,f). At an equimolar ratio of actin and cofilin, the addition of either tropomyosin did not change the fluorescence signal in the presence of cofilin-2 and only slightly restored it in the presence of cofilin-1 (Figure 5d,g).

## 4. Discussion

### 4.1. Effects of Different Cofilin Isoforms on F-Actin Covered by Tpm4.1 and Tpm4.2

The *TPM4* gene produces only two Tpm isoforms: Tpm4.1 and Tpm4.2 [42]. They belong to two different groups of tropomyosins divided according to their molecular weights: Tpm4.1 belongs to the HMW isoform group, while Tpm4.2 belongs to the LMW group. The difference in the protein structure is in the N-terminal part of the molecule. The mRNA molecule producing Tpm4.1 starts from **1a2b** exon, while that producing Tpm4.2 starts from **1b** [42]. Although there are differences between them, these two isoforms have a similar ability to determine cofilin’s functions. In fact, in all the experiments conducted, the difference between the isoforms was either absent or minimal.

Both isoforms increased the viscosity of F-actin solutions in the presence of cofilin-1 and cofilin-2 (Figure 1, Table 1). The viscosity values correlated with the size of the actin filaments, suggesting that following cofilin treatment, the actin filaments were longer in the presence of these Tpm isoforms than in their absence. At the same time, the protective effects of Tpm4.1 and Tpm4.2 are controversial. On the one hand, these isoforms exert a protective effect against cofilin’s severing/depolymerization activity by increasing the length of the actin filaments in the presence of cofilin. One the other hand, if we look at the relative values, the observed protective effect is exclusive to Tpm4.2, since the length of the actin filaments is initially longer in the presence of these Tpm isoforms. Interestingly, Gateva et al. also found that Tpm4.2 was largely ineffective in protecting actin filaments from cofilin severing, which is consistent with our data [55]. At the same time our data showed that Tpm4.1 affects the actin filament length rather than cofilin’s severing/depolymerizing activity.

The results of the viscosity experiments were in good agreement with the electron microscopy results obtained for Tpm4.2. We found a difference in the actin length proportions after cofilin-1 treatment in the presence and absence of Tpm4.2. Most actin filaments were longer in the presence of Tpm4.2. This confirms our suggestion that the viscosity depends on the actin filaments’ length.

The ability of cofilins to bind to actin filaments in the presence of these isoforms is remarkable. Tpm4.1 and Tpm4.2 did not actually interfere with the binding of cofilin to actin filaments (Figure 2). Notably, both cofilin isoforms bound well to the actin surface in the presence of these isoforms, but their ability to induce structural rearrangements in the actin filament was different. Changes in the actin filament conformation induced by cofilins in the presence of Tpm isoforms were detected using the rhodamine–phalloidin fluorescence decay. Phalloidin and cofilin bound with actin filaments at different sites [22], working like antagonists. Binding of phalloidin inhibits binding of cofilin and vice versa [72]. Therefore, the binding of cofilin to actin depends on both the cofilin and phalloidin concentrations. At high cofilin concentrations, cofilin displayed the displacement of phalloidin from the F-actin filaments. Taking into account that they have different binding sites, such displacement reflects conformational changes in the actin filament. Both Tpm isoforms (Tpm4.1 and Tpm4.2) did not interfere with this process. Cofilin-2 resulted in a more rapid displacement of phalloidin from the actin filament surface, suggesting that it may induce more pronounced changes in the actin filament conformation. Since cofilin-2 is primarily produced in muscle tissue, this may be important for its functions. These tissues express Tpm isoforms that preferentially protect actin filaments from the severing activity of cofilin. Perhaps the enhanced ability of cofilin-2 to induce structural rearrangements in filamentous actin could contribute to more efficient severing. It is also worth noting that in our experiments we used skeletal muscle actin, and it is possible that cofilin-2 interacts better with muscle actin isoforms than cofilin-1, which is expressed predominantly in non-muscle tissues.

Interesting data were obtained when assessing the effect of cofilin on the polymerization of actin filaments in the presence of isoforms Tpm4.1 and Tpm4.2 (Figure 5a). Both isoforms tended to decrease the rate of actin polymerization since actin’s main polymerization curves were lower than in the absence of Tpm; nevertheless, the standard deviation spreads obtained from the repetitions for all the curves intersected. The decrease in the rate of actin polymerization in the presence of Tpm is confirmed in the literature. Most Tpm isoforms inhibit the rate of spontaneous actin polymerization [56,73]. In turn, both isoforms of cofilin accelerated the polymerization of actin filaments at a concentration ratio of cofilin/actin equal to 1:10. However, their combined action with Tpm turned out to be much more complicated at this concentration ratio (Figure 5b,e). In the initial stages of polymerization, the effect of the Tpms dominated over the effect of the cofilin molecules. The polymerization rate was lower than that obtained for actin alone. However, in the late stages of polymerization, cofilin accelerated actin polymerization, which allowed the curves to reach a plateau earlier. Since Tpm4.1 and Tpm4.2 exhibit almost no interference with the binding of cofilin to filamentous actin and do not inhibit its severing/depolymerizing activity, this effect may seem contradictory. This can probably be explained by the fact that in the early stages of polymerization, cofilin binds to the actin filament and does not exhibit severing activity. When the filament length begins to increase, cofilin severs the actin filament, leading to the generation of more primers for actin filament growth. At higher ratios of cofilin to actin, both cofilins reduced the rate of polymerization (Figure 5c,f). This result is in good agreement with the data obtained in experiments on actin polymerization in the presence of different concentrations of cofilin [74]. The presence of Tpm in such a system only enhanced the effect of the cofilin molecules.

It is especially important to note that cofilin is capable of quenching the pyrene fluorescence when measuring the rate of actin polymerization. At cofilin ratios lower than the actin concentrations, this effect is limited. In particular, Umeki and co-authors established that when the concentration of cofilin is two times lower than that of actin, the quenching reaches no more than 30% [75]. However, if the concentration of actin is comparable to the concentration of cofilin, this leads to almost complete quenching of the pyrene fluorescence [76] (Figure 5d,g). Thus, the data obtained with this cofilin/actin ratio cannot be used to determine the influence on the polymerization rate. Dedova and co-authors showed that quenching of the pyrene fluorescence at an equal cofilin/actin ratio is associated with structural rearrangement in actin filaments during cofilin binding [76]. These data (Figure 5d,g) are in good agreement with the results for the rhodamine–phalloidin decay (Figure 3), where the effect of cofilin-2 was more pronounced than that of cofilin-1.

### 4.2. Determination of Cofilin’s Function by Tpm4.1 and Tpm4.2 in Comparison with Other Tpm Isoforms

There is a huge diversity of Tpm isoforms that are expressed in various human tissues and cell compartments. Some evidence has been obtained that several of these isoforms affect the functional characteristics of cofilin. In this section, we compare the effects of different Tpm isoforms on the functional characteristics of cofilin with the effects of Tpm4.1 and Tpm4.2 that we studied.

Since the structure of tropomyosin genes is similar, isoforms that have the same set of exons during expression can be found in different genes. In this regard, it is interesting to compare the effects of Tpm1.6 and Tpm4.1, which have the same set of exons: **1a2b6b9d**. The properties of Tpm1.6 and its ability to determine the functions of cofilin-1 were discussed in detail in a paper by Ostrowska et al. [56]. Surprisingly, these isoforms differed completely in their effect on the action of cofilin-1 on actin filaments. Thus, 1.6 protected against the depolymerizing action of cofilin-1, while 4.1 had no effect (Figure 1). There were also significant differences in the dissociation of Tpm from the actin surface between these isoforms. We observed a 50% displacement of Tpm4.2 in the presence of cofilin-1 at a 1:2 ratio to F-actin (Figure 2), whereas a 4-fold-higher ratio was required to achieve similar results for Tpm1.6 [56]. These isoforms also differ in their effects on F-actin polymerization in the presence of cofilin-1. Both isoforms inhibit the rate of spontaneous actin polymerization in the absence of cofilin-1. In the presence of cofilin-1, however, the polymerization rate reached the values obtained for the actin control in the case of Tpm1.6 [56], while the effect of Tpm4.1 remained dominant in the initial stages of polymerization. All this suggests that the presence of precise substitutions in the production of these Tpm isoforms from different genes is crucial in determining their functions in relation to cofilin.

By analogy with the pair of Tpm4.1 and Tpm1.6, it is interesting to compare Tpm3.2 and Tpm4.2, which are also expressed with the same set of variable exons: **1b6b9d**. They have both similar and different properties in terms of their determination of cofilin-1’s functions. Both isoforms were effectively displaced from the surface of the actin filament [56] (Figure 2). Their effects on G-actin polymerization in the presence of cofilin-1 were different. Tpm3.2 slightly increased the polymerization rate in the presence of cofilin-1 in comparison with that of G-actin alone [56], while Tpm4.2 decreased it (Figure 5). The effect of Tpm3.2 and Tpm4.2 on cofilin-1’s severing/depolymerizing action on F-actin also differed. Tpm3.2 promoted it [56], whereas Tpm4.2 slightly protected actin filaments from the action of cofilin-1 (Figure 1 and Figure 4).

It is also interesting to compare Tpm4.1 and Tpm4.2 with other tropomyosin isoforms. All Tpm isoforms can be divided into groups with respect to their effect on the action of cofilin: isoforms that inhibit the action of cofilin and those that promote it. It is generally accepted that most HMW Tpms inhibit the severing/depolymerizing action of cofilin-1 and protect the actin filament. This has been shown for Tpm1.1, Tpm3.12, Tpm1.6, and Tpm1.7 [55,56,58,59]. Tpm4.1 differs from them in this regard. At the same time, LMW Tpm isoforms such as Tpm1.12, Tpm3.2, and Tpm3.4 promote the severing action of cofilin [55]. At first glance Tpm4.2 can be assigned to this group since it allows cofilin molecules to easily bind to the surface of the actin filament and only slightly inhibits their severing activity. However, in the presence of Tpm4.2, following treatment with cofilin-1, the actin filaments remain longer due to the action of Tpm4.2 than in its absence (Figure 4). Thus, both of these isoforms (Tpm4.1 and Tpm4.2) differ from most representatives of their groups, which makes them unique in terms of their determination of cofilin’s functions.

### 4.3. Potential Role of Tpm4.1 and Tpm4.2’s Interaction with Cofilin Isoforms

The cytoskeleton’s dynamics rely heavily on tropomyosin and cofilin, which are actin-binding proteins, making their combined action of great importance. Both cofilin isoforms are expressed in all tissues, just in different amounts [33]. It is generally accepted that cofilin-2 and cofilin-1 coexist in the muscle tissue [39]. In the non-muscle tissue cofilin-1 is the main isoform [37]. The production of Tpm isoforms that are expressed by the *TPM4* gene has also been found in skeletal tissue, as shown in fish, and non-muscle tissues [60,61,63]. In some ways, this makes these proteins unique to study, as they can potentially be found in almost any cell.

It is even more interesting that Tpm4.1 and Tpm4.2 have almost no effect on the binding of cofilin to actin filaments. This circumstance makes it difficult to predict the effect of these forms on the action of cofilin. On the one hand, cofilin can bind in large quantities to the surface of the actin filaments in the presence of these isoforms. This should lead to a rapid reorganization of the actin cytoskeleton in this region. On the other hand, downregulation leads to the disruption of cell adhesion and promotes the invasion of cancer cells, which indicates its important role in stabilizing actin structures. At the same time, Tpm4.2 has been shown to have an inactivating effect on cofilin, accompanied by its phosphorylation and a decrease in its binding to actin. This effect may be associated with the regulation of the expression of kinases and phosphorylases which regulate the action of cofilin and inhibit it in response to increased Tpm4.2 expression, as suggested by Suchowerska et al. [63]. Inactivated cofilin can also bind to the actin filament in the presence of these isoforms and wait for an activating signal, which means that Tpm4.1 and Tpm4.2 can fine-tune actin structures depending on the processes occurring in the cell.

It is also interesting to consider the effect of Tpm4.1 and Tpm4.2 on the action of cofilin in relation to its concentration. It has been shown that sufficiently high concentrations of cofilin-1 can be achieved in the cell. This may lead to the appearance of zones where the local concentration of cofilin is comparable to that of actin. Saturating concentrations of cofilin, contrary to their usual effect, are well known to stabilize the actin filament. The presence of isoforms that can be easily removed from the surface of the actin filament and do not interfere with the binding of cofilin to it can provide such saturation. Perhaps, this may also have functional significance for the dynamics of the actin cytoskeleton. Such Tpm isoforms can also help cofilin to reach its effective concentration. Tropomyosin isoforms that efficiently block access to the actin filament almost completely should inhibit the action of cofilin and form elements with long-term stability in the cell. On the other hand, tropomyosin isoforms that do not compete so strongly with cofilin for binding may help cofilin reach an effective concentration, which is especially necessary for severing the actin filament.

Despite a considerable number of studies on the dynamics of the actin cytoskeleton under the influence of cofilin in the presence of Tpm, there are still many gaps and contradictions. Studying more tropomyosin isoforms, along with varying conditions and the addition of different actin-binding proteins, may help clarify this issue.

## 5. Conclusions

Cofilin and tropomyosin are two important participants in the regulation of the actin cytoskeleton’s dynamics. In this work, we studied the mutual influence of two Tpm isoforms, Tpm4.1 and Tpm4.2, and two cofilin isoforms, cofilin-1 and cofilin-2, on their functional properties. Tpm4.1 and Tpm4.2 had similar effects on the cofilin activity and did not show differences in these effects depending on the isoforms of the cofilin molecules. It is likely that in the presence of these Tpm isoforms, cofilin-1 and cofilin-2 have a similar pattern of activity. Cofilins bound well to the surface of the actin filaments in the presence of Tpm4.1 and Tpm4.2. In this case, the depolymerizing activity of the cofilin molecules did not increase and even slightly decreased in the presence of Tpm4.2. This makes the role of Tpm4.1 and Tpm4.2 in determining the activity of cofilin molecules confusing and controversial. It is quite possible that these isoforms can influence the length of the actin filaments under the action of cofilin molecules. Thus, Tpm4.2 led to an increase in the length of the actin filaments under the action of cofilin-1 in comparison with the length of the bare actin filaments after treatment with cofilin-1.

## Figures and Tables

**Table 1:** Effect of cofilins on the F-actin solution viscosity in the presence of Tpm4.1 or Tpm4.2.

	W/o Cofilins	With cof-1		With cof-2	
	Δη_0_^1^, mPa·s	Δη^2^, mPa·s	Δη/Δη_0_, %	Δη^2^, mPa·s	Δη/Δη_0_, %
F-actin	0.66 ± 0.04	0.42 ± 0.04	64 ± 8	0.38 ± 0.03	58 ± 7
+Tpm4.1	0.77 ± 0.04	0.57 ± 0.03	74 ± 7	0.52 ± 0.04	68 ± 7
+Tpm4.2	0.77 ± 0.04	0.62 ± 0.04	81 ± 7	0.56 ± 0.04	73 ± 7

All values are presented as mean ± confidence interval *(p* = 0.95). ^1^Δη_0_—excess solution viscosity over the buffer viscosity in absence of cofilins; ^2^Δη—excess solution viscosity over the buffer viscosity.

## Data Availability

All data is presented in the article.

## Supplementary Materials

The following supporting information can be downloaded at: https://www.mdpi.com/article/10.3390/biom15081206/s1, File S1: Original images.

## Abbreviations

The following abbreviations are used in this manuscript:

HMW	High molecular weight
LMW	Low molecular weight
cof-1	Cofilin-1
cof-2	Cofilin-2
Tpm	Tropomyosin
