# Supplementary figures and images for: Role of Tpm Isoforms Produced by the TPM4 Gene in the Regulation of Actin Filament Dynamics by Cofilin
Source: Biomolecules. 2025 Aug 21;15(8):1206. doi: 10.3390/biom15081206 (PMC12384860; doi:10.3390/biom15081206)

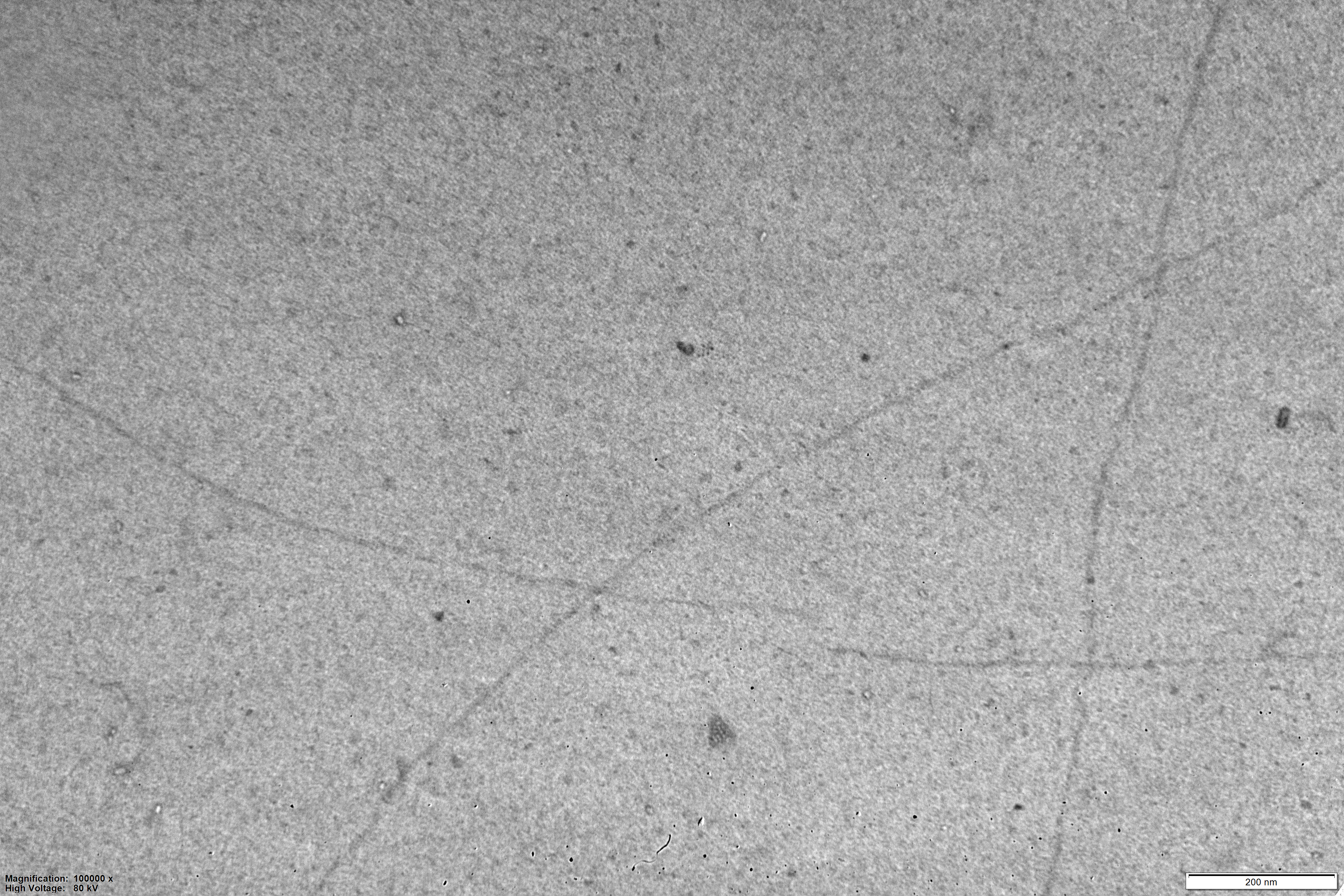

Supplement: Supplementary file 1 [file biomolecules-15-01206-s001.zip › Image_12405_F-actin_cof_Tpm4.2.tif]

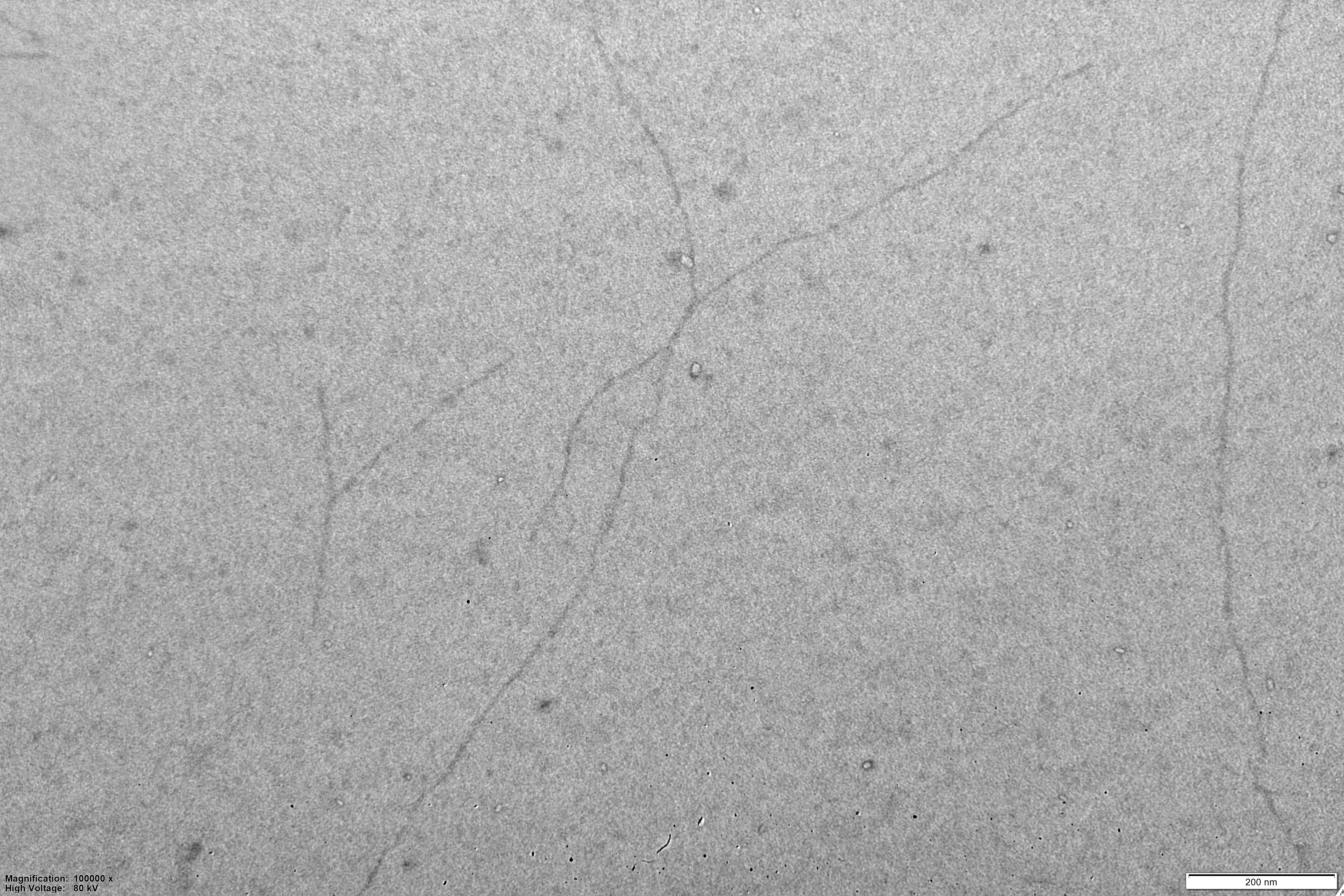

Supplement: Supplementary file 1 [file biomolecules-15-01206-s001.zip › Image_12428_F-actin_cof1.tif]

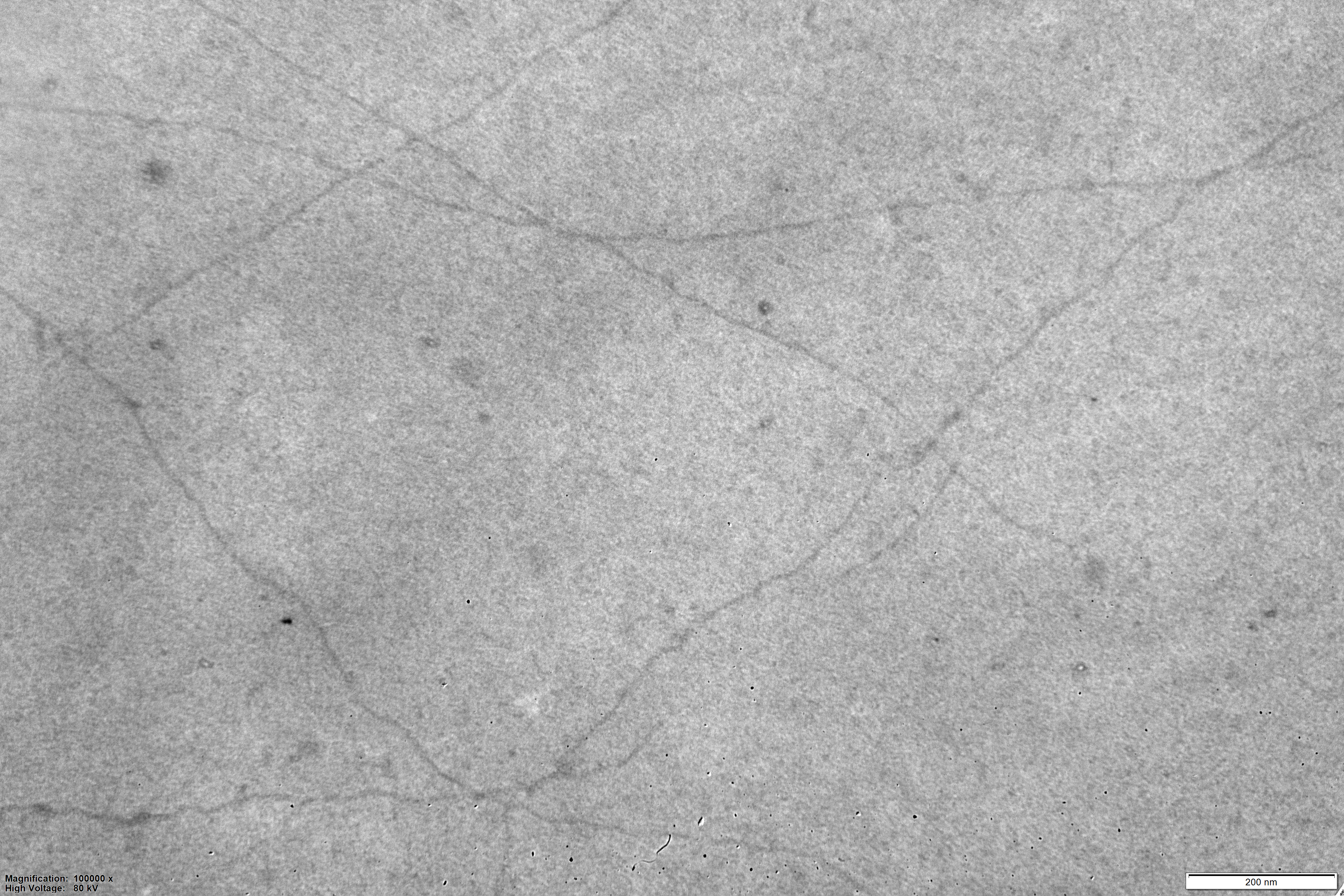

Supplement: Supplementary file 1 [file biomolecules-15-01206-s001.zip › Image_12462_F-actin_Tpm4.2.tif]

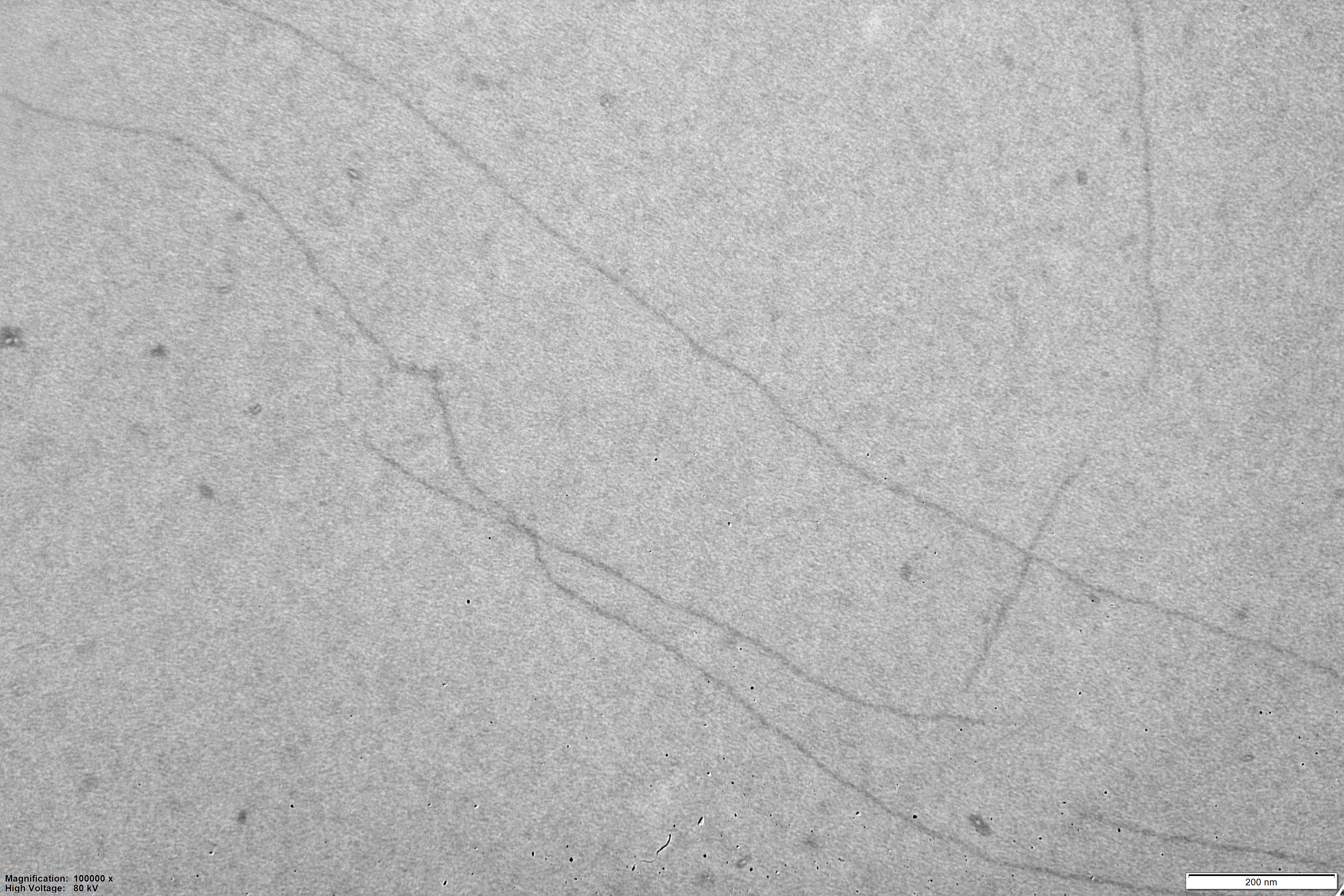

Supplement: Supplementary file 1 [file biomolecules-15-01206-s001.zip › Image_12477_F-actin.tif]
